# On the Pharmaceutical Preparation of Carbonate of Iron

**Published:** 1830-10-01

**Authors:** 


### LXIII.

### On the Pharmaceutical Preparation of the Precipitated Carbonate of Iron. By Mr. Thomas Clark.

The last number of our Glasgow cotemporary contains a short paper under the above title, which we deem it proper to notice. The British Pharmacopœias direct watery solutions of sulphate of iron and subcarbonate of soda to be mixed, and the resulting precipitate to be collected by a filter and dried. The precipitate, at first is white, but soon becomes of a dark green colour, and very bulky in substance. When drained of the water, it is still found to be bulky. Exposed to the air the colour changes to a rusty yellow, the effect of oxygen. A decomposition is produced, according to our author in the following manner.

"The precipitated carbonate of iron consists of carbonic acid combined with the *black* oxide of iron; which black oxide readily combines with more oxygen forming the *red* oxide; but as the red oxide cannot, like the black oxide, retain carbonic acid in combination, this acid flies off; so that, in the yellow matter alluded to, an additional dose of oxygen has taken the place before held by carbonic acid. The yellow colour is owing to the red oxide existing in combination with water, or, (to use the language of modern chemistry,) as *a hydrate;* and the yellow colour is changed to red whenever we apply so much heat as will drive off the combined water. Then the red oxide of iron, or colcothar of vitriol, alone remains."

The consequence is that what is sold in the shops for precipitated carbonate of iron contains no more than a trace of that substance — and is frequently nothing more than colcothar of vitriol. This colcothar is not less different (Mr. C. observes) from carbonate of iron in its medicinal than in its chemical properties.

"I have seen patients, of different ages and sexes, swallow, for a fortnight, at the rate of half an ounce a-day, of colcothar of vitriol, without producing any apparent effect, except that their stools were coloured by the powder to a reddish hue, indicating that it had passed through the body unaltered; whereas I have seen a healthy man made sick by a dose of a quarter of a drachm of genuine carbonate of iron, and made to pass in consequence dark greenish black stools for two days after; and I have seen similar effects produced on patients who had been unaffected by colcothar of vitriol. The sickness, however, is not produced after the first or second day."

These observations deserve the attention of the profession in these days when carbonate of iron is so much in use. We give the remaining part of the paper in the author's own words.

"From the preceding observations, it is easy to gather, that the two defects to be avoided, are exposure to air and exposure to heat. Both of these defects I propose to avoid, by forming the precipitated carbonate into an electuary, thus—

Take of sulphate of iron and subcarbonate of soda, each eight ounces. Pound each salt, and dissolve them separately in warm water. If necessary filter. Being filtered and cool, mix the solutions in a deep vessel, capable of holding one or two gallons of water, which fill up cold. Stir—let subside—and then decant the clear liquor from the precipitate. Fill up again with water, and likewise again decant; and repeat this operation two or three times, so as to separate the soluble salts. Next put the precipitate on a filter of cotton or linen cloth, supported by a square frame. When the water has ceased to pass, gather into one hand the edges of the filter, so as to make it a sort of bag, and with the other twist it round from the holding hand downwards, so as to squeeze out the remaining water. The precipitate will now have the appearance of clay, too soft for moulding. With soft sugar and aromatic powder, in suitable proportions, make it into an electuary.

Thus we obtain a carbonate of iron, uniform in its properties, hardly deteriorated by the process it undergoes, and little liable to change by keeping.

The precipitated carbonate of iron, while yet moist, is soluble in carbonic acid. Hence a teaspoonful of the above electuary is soon dissolved in a glass of ginger beer, except the aromatic powder it contains. It may be asked, therefore, whether an eligible medicine might not be obtained as follows:—Having filled a dozen of bottles with ginger beer, divide among them the precipitate from an ounce of sulphate of iron, and an ounce of subcarbonate of soda: then cork and set them aside, as usual, till they be ready. I presume that the production of carbonic acid, by the fermenting process, would go on as usual, and that when drawn in due time, we would find the carbonate of iron entirely dissolved in the ginger beer."

